# Supplementary material for: Widespread human exposure to ledanteviruses in Uganda: A population study
Source: PLoS Negl Trop Dis. 2024 Jul 8;18(7):e0012297. doi: 10.1371/journal.pntd.0012297 (PMC11257405; doi:10.1371/journal.pntd.0012297)
Supplement: S1 Table — (DOCX) [file pntd.0012297.s001.docx]

|  | |  |
| --- | --- | --- |
| **Table S1:** Assays employed in the acute febrile illness study (AFI) | | |
| Pathogen | Assay | |
| Leptospirosis | Leptospirosis IgM lateral flow assay (Lifeassay diagnostics, Cape Town, SA) | |
| Brucellosis | Brucella IgM lateral flow assay (Lifeassay diagnostics, Cape Town, SA) | |
| Malaria | Thick and thin blood films  Rapid 1-2-3 malaria HEMA Express rapid diagnostic kit (Miramar, Florida, USA.) | |
| Dengue virus (acute samples) | Dengue NS1 Ag ELISA (Standard Diagnostics Inc. Kyonggido, Korea) | |
| Dengue virus (convalescent samples) | Dengue IgM ELISA (Standard Diagnostics Inc. Kyonggido, Korea) | |
| Chikungunya | Chikungunya IgM ELISA (Standard Diagnostics Inc. Kyonggido, Korea) | |
| O’nyong nyong | In-house IgM ELISA developed at UVRI | |
| West Nile Virus | West Nile virus IgM Capture ELISA (FOCUS Diagnostics Cypress, CA) | |
| Yellow Fever Virus | In-house ELISA developed at UVRI | |
| Typhoid Fever | Acute and convalescent IgM Tubex serology (IDL Biotech, Bromma, Sweden). | |
| Rickettsioses | Rickettsia indirect fluorescent (IFA) IgG assay (Focus Diagnostics Cypress, CA) | |
