# Supplementary material for: Widespread human exposure to ledanteviruses in Uganda: A population study
Source: PLoS Negl Trop Dis. 2024 Jul 8;18(7):e0012297. doi: 10.1371/journal.pntd.0012297 (PMC11257405; doi:10.1371/journal.pntd.0012297)
Supplement: S2 Table — (DOCX) [file pntd.0012297.s002.docx]

| **Table S2.** Genomic distance between LDV-Uganda and LDV-Senegal (DakHD763/KM205006) | | | | | | | |
| --- | --- | --- | --- | --- | --- | --- | --- |
| Region | N | P | M | G | U | L | Intergenic |
| Nucleotide p-distance | 5.25 | 6.84 | 5.68 | 5.75 | 9.23 | 6.38 | 5.17 |
| Amino acid p-distance | 0.24 | 3.86 | 1.87 | 2.62 | 14.06 | 2.35 | - |
