# Supplementary material for: Widespread human exposure to ledanteviruses in Uganda: A population study
Source: PLoS Negl Trop Dis. 2024 Jul 8;18(7):e0012297. doi: 10.1371/journal.pntd.0012297 (PMC11257405; doi:10.1371/journal.pntd.0012297)
Supplement: S3 Table — (DOCX) [file pntd.0012297.s003.docx]

| **Table S3: number of rodents by species captured for serum mNGS in Adumi subcounty.** | |
| --- | --- |
| *Aethomys kaiseri* | 17 |
| *Arvicanthus niloticus* | 13 |
| *Crocidura spp.* | 57 |
| *Lemniscomys striatus* | 6 |
| *Mus minutoides* | 7 |
| *Mastomys spp.* | 26 |
| *Rattus rattus* | 64 |
| *Gerbilliscus validus* | 8 |
| *Taterillus emini* | 2 |
| *Thamnomys spp.* | 2 |
| *Zelotomys hildegardeae* | 3 |
