# Supplementary material for: Widespread human exposure to ledanteviruses in Uganda: A population study
Source: PLoS Negl Trop Dis. 2024 Jul 8;18(7):e0012297. doi: 10.1371/journal.pntd.0012297 (PMC11257405; doi:10.1371/journal.pntd.0012297)
Supplement: S4 Table — (DOCX) [file pntd.0012297.s004.docx]

| **Table S4: blastx results of *de novo* contigs from sequencing of blood from *Mastomys erythroleucus*** | | | | | |
| --- | --- | --- | --- | --- | --- |
| Contig name | Accession of blastx hit | Protein | Virus | Identity (%) | Length (AA) |
| merged_contig3927 | YP_009361873 | polymerase | LDV | 78.2 | 271 |
| merged_contig4193 | YP_009361873 | polymerase | LDV | 87.9 | 471 |
| merged_contig5160 | YP_009361873 | polymerase | LDV | 67.9 | 153 |
| merged_contig5201 | YP_009362198 | glycoprotein | KEUV | 76.5 | 421 |
| merged_contig5497 | YP_009361873 | polymerase | LDV | 86.4 | 140 |
| merged_contig6266 | YP_009361873 | polymerase | LDV | 76.8 | 69 |
| merged_contig7735 | YP_009362195 | nucleoprotein | KEUV | 84.3 | 268 |
| merged_contig8264 | YP_009361873 | polymerase | LDV | 72.1 | 226 |
| merged_contig10327 | YP_009361873 | polymerase | LDV | 84.1 | 531 |
| idba-121_14222 | YP_009362196 | phosphoprotein | KEUV | 63.9 | 72 |
| idba-121_8592 | YP_009362194 | polymerase | LDV | 59.6 | 47 |
| spades_37480 | YP_009361868 | nucleoprotein | LDV | 70.8 | 96 |
| spades_75880 | YP_009361869 | phosphoprotein | LDV | 49.4 | 79 |
