# Supplementary material for: Widespread human exposure to ledanteviruses in Uganda: A population study
Source: PLoS Negl Trop Dis. 2024 Jul 8;18(7):e0012297. doi: 10.1371/journal.pntd.0012297 (PMC11257405; doi:10.1371/journal.pntd.0012297)
Supplement: S5 Table — (DOCX) [file pntd.0012297.s005.docx]

| **Table S5: pairwise amino acid distance between the *Mastomys erythroleucus-*associated ledantevirus and other phylogroup B ledanteviruses** | | | | |
| --- | --- | --- | --- | --- |
| Virus (accession) | Gene | | | |
|  | N | P | G | L |
| KEUV (KM205021) | 21.45 | 50.00 | 24.03 | 21.01 |
| LDV (KM205006) | 21.18 | 46.15 | 24.57 | 18.36 |
| VAPV (MG021441) | 36.46 | 58.19 | 37.71 | 29.37 |
| KCV (KM204992) | 50.40 | 70.39 | 47.79 | 36.46 |
