# Supplementary figures and images for: Widespread human exposure to ledanteviruses in Uganda: A population study
Source: PLoS Negl Trop Dis. 2024 Jul 8;18(7):e0012297. doi: 10.1371/journal.pntd.0012297 (PMC11257405; doi:10.1371/journal.pntd.0012297)

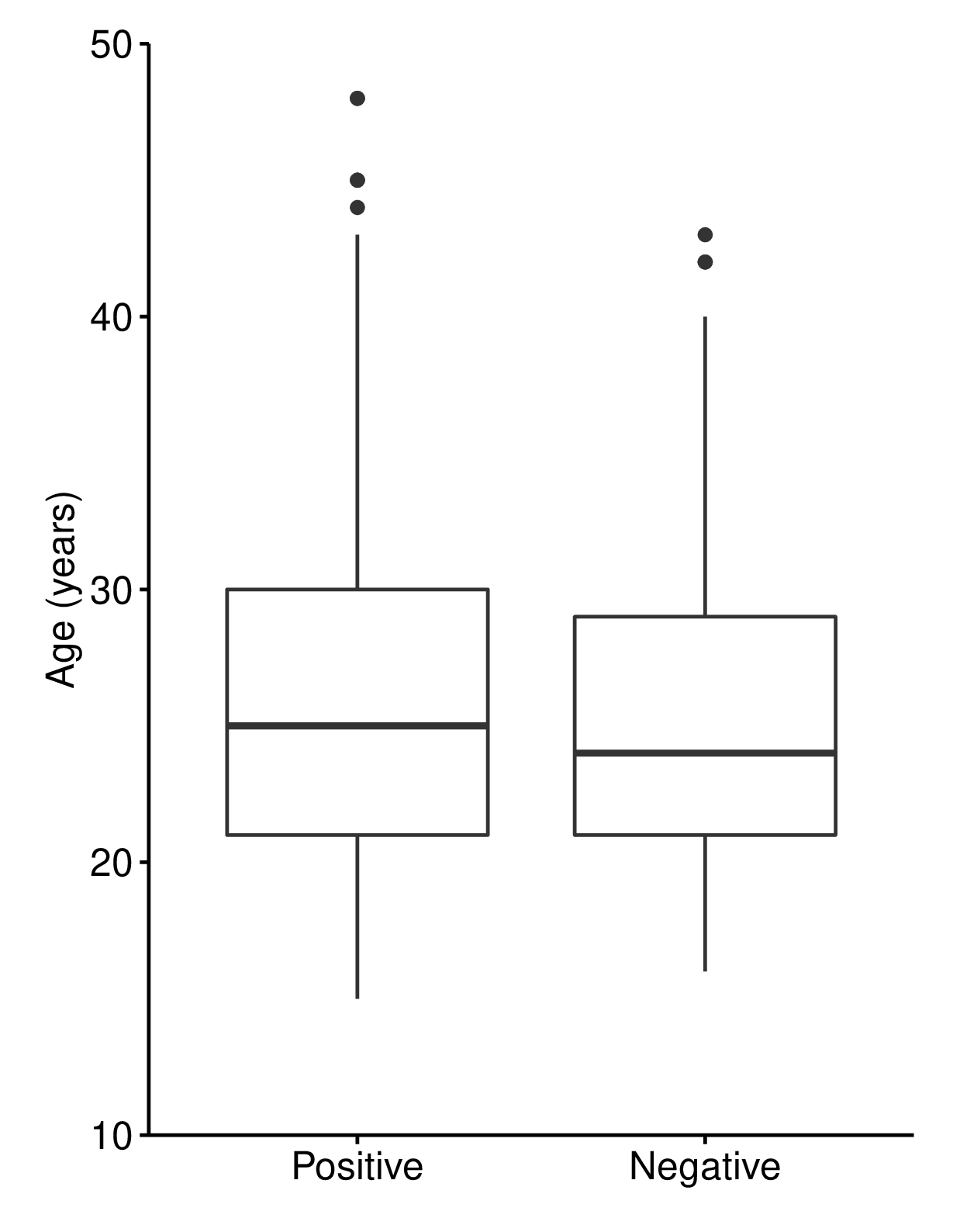

Supplement: S1 Fig — Age by seroprevalence as determined by LDV-G ELISA for individuals in the ANC-2016 cohort (n = 997). Median age in the seropositive group was 25 years (n = 451, IQR = 21–30) compared to 24 in the seronegative group (n = 546, IQR = 21–29), Mann-Whitney U, p = 0.016. Centre bars represent the median, box edges the IQR, vertical lines the range (1.5 times the IQR from the upper and lower quartile), and points the outliers. (TIF) [file pntd.0012297.s012.tif]

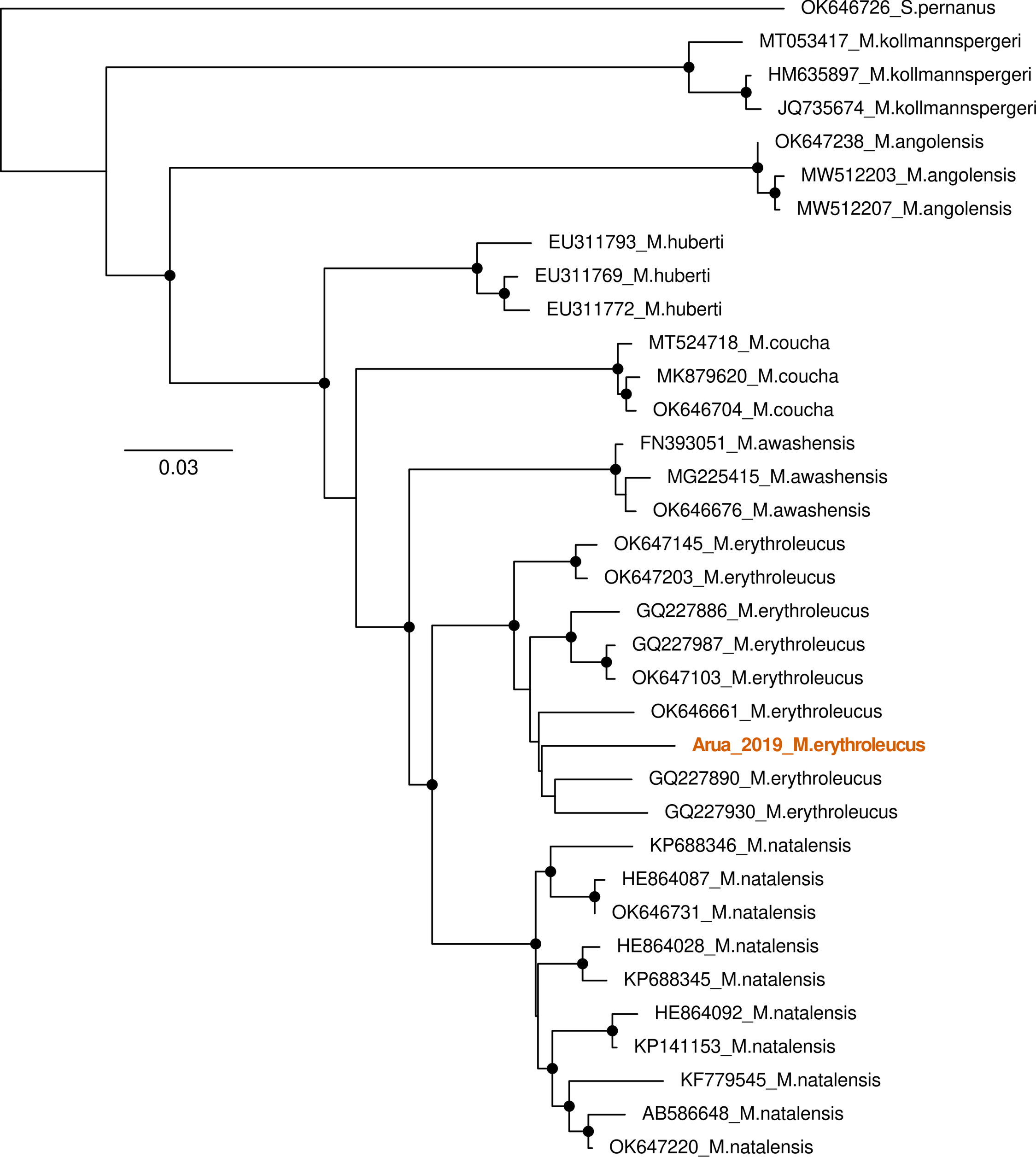

Supplement: S2 Fig — Maximum likelihood phylogeny of mitochondrial cytochrome B sequences derived from representative Mastomys species. The sequence derived from the animal in which the Mastomys erythroleucus associated ledanteviruses was detected is indicated in orange. Serengetimys pernanusis is included as an outgroup. Circles indicate nodes with bootstrap support >70. (TIF) [file pntd.0012297.s013.tif]

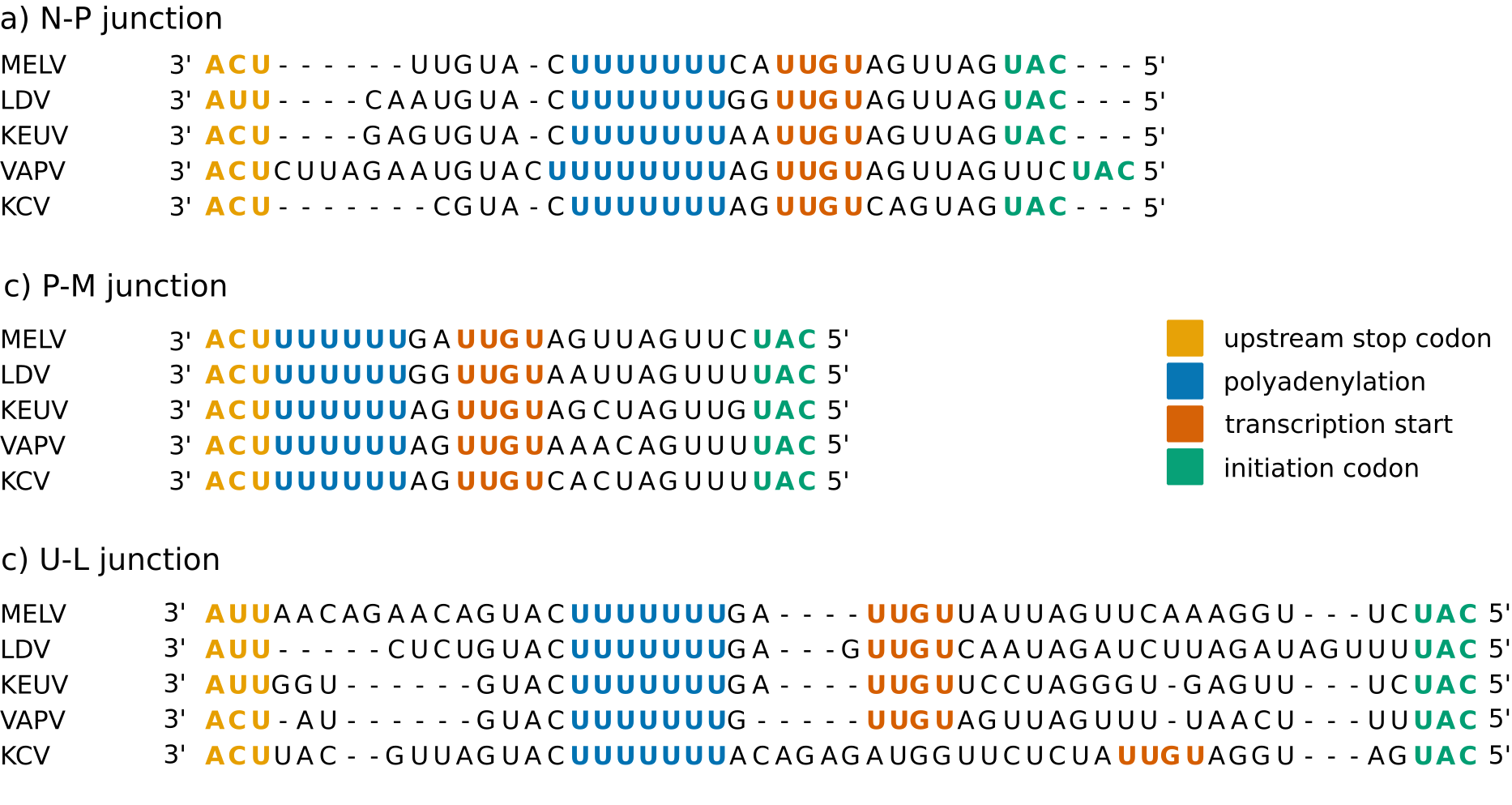

Supplement: S3 Fig — Genomic gene junction regions of MELV compared with other group B ledanteviruses demonstrating the presence of conserved rhabdovirus intergenic polyadenylation and transcription initiation signals. a) nucleoprotein–phosphoprotein junction. b); phosphoprotein matrix protein junction. c) accessory protein–RNA dependant RNA polymerase junction. MELV; Mastomys erythroleucus—associated ledantevirus, LDV; Le Dantec virus, KEUV, Keuraliba virus, VAPV; Vaprio virus, KCV; Kern Canyon virus. (TIF) [file pntd.0012297.s014.tif]
