# Supplementary material for: Widespread human exposure to ledanteviruses in Uganda: A population study
Source: PLoS Negl Trop Dis. 2024 Jul 8;18(7):e0012297. doi: 10.1371/journal.pntd.0012297 (PMC11257405; doi:10.1371/journal.pntd.0012297)
Supplement: Attachment — Submitted filename: Response_to_reviewers_LDV_PLOSNTD.pdf [file pntd.0012297.s015.pdf]

This manuscript was previously submitted to PLOS pathogens and transferred to PLOS Neglected Tropical Diseases without review. The following change has been made to the manuscript:

An additional reference has been added: Kigozi BK, Kharod GA, Bukenya H, Shadomy SV, Haberling DL, Stoddard RA, et al. Investigating the etiology of acute febrile illness: a prospective clinic-based study in Uganda. BMC Infectious Diseases. 2023;23(1):411.
