# Supplementary material for: Widespread human exposure to ledanteviruses in Uganda: A population study
Source: PLoS Negl Trop Dis. 2024 Jul 8;18(7):e0012297. doi: 10.1371/journal.pntd.0012297 (PMC11257405; doi:10.1371/journal.pntd.0012297)
Supplement: Attachment — Submitted filename: response_to_reviewers.pdf [file pntd.0012297.s017.pdf]

Medical Research Council
University of Glasgow
Centre for Virus Research

Thomson lab
MRC-University of Glasgow
Centre for Virus Research
Garscube Campus
464 Bearsden Road
Glasgow G61 1QH
Scotland (UK)

T: +44 (0)141 330 2928
M: +44 7870 653 680
Fax: +44(0) 141 330 2271
W: www.cvr.ac.uk

19th April 2024

Dear colleagues,

Re: Widespread human exposure to ledanteviruses in Uganda: a population study.

Many thanks for your invitation to submit a revised version of our manuscript. We would like to thank all three expert reviewers for their insightful comments and suggestions. Their input has strengthened the paper, and we thank them for their time and effort.

We have significantly rewritten the discussion in line with the suggestions of reviewer 1, whilst also addressing the comments and suggestions of all three reviewers.

Please find below a point-by-point response to the comments made by the reviewers below.

Yours faithfully,

Dr James Shepherd

Professor Emma Thomson

MRC-University of Glasgow Centre for Virus Research

Reviewer #1: This intriguing manuscript describes a new case of ledantec virus (LDV) infection in humans (at least the third documented case, not the second, as the authors claim) and a serosurvey of antibodies to LDV and its relatives in Uganda. The serologic assays are impressive and the authors should be commended for their efforts to investigate human infection.

Unfortunately, the manuscript fails to place its findings in an appropriate scientific, historical, ecological or epidemiological context. There is a significant literature on these viruses, including in Uganda, which the authors do not cite or merely cite cursorily. As a result, many of the main conclusions drawn by the authors are deeply in error, based on known facts from the published literature.

The authors should "start from scratch." They should step back and familiarize themselves with the fascinating history and biology of the ledanteviruses. They should read and understand all the research on these viruses that has preceded their work (it's a rich literature but not intractably extensive). They should then rewrite the manuscript entirely, structuring it around the current state of knowledge. An incomplete list of essential features of this restructuring includes:

1) Where, in nature, other ledanteviruses have been found.

2) A detailed review of the strong evidence showing that ledanteviruses are vector-borne, including which vectors have been associated with which viruses. This is true even for ledanteviruses for which no vector has ever been identified.

3) A review of how ledanteviruses are thought to be transmitted among mammalian hosts by vectors, maintained in mammalian hosts by vectors, and occasionally transmitted to humans by those vectors, including documented examples going back many decades.

4) Which ledanteviruses have previously been discovered in Uganda, where, when, and under what circumstances. The authors do not cite a number of key papers from several research groups about other ledanteviruses in Uganda, including zoonotic transmission.

5) Which ledanteviruses have previously been discovered in other African countries. Note Kenya, for example, where Mt Elgon Bat virus was found – Mt. Elgon lies on the border between Uganda and Kenya.

6) Which ledanteviruses have previously been discovered in countries outside of Africa (ledanteviruses are found around the world), and how our knowledge of these viruses in aggregate paints a fairly clear picture of the biology, ecology and epidemiology of these viruses globally.

The authors are strongly advised to seek the expert opinion of a specialist in ledanteviruses as they rewrite their manuscript. This would help the authors form more accurate conclusions about their own data.

We have extensively rewritten the manuscript, in particular the discussion as suggested expanding on the history and background of the ledanteviruses with a focus on key papers relevant to this study. Given this is primarily a research article we have attempted to balance the need for a review of the relevant literature whilst focusing on interpretation of the findings presented in the article.

Line 22 and 179. This appears to be a cross-sectional sample. Please delete "a cohort of."

We have removed this text.

Line 34. Change "pandemics" to "epidemics" to avoid alarmist language.

We have changed the language as suggested.

Line 34-36. The grammar of this sentence is confusing. What is the hotspot? Significant gaps? Africa? Please re-write sentence to be clearer.

We have removed this sentence.

Line 46. "Environmental reservoir" is not accurate. Either delete "environmental" or change to "animal reservoir."

We have changed the text as suggested.

Lines 49-66. The opening paragraphs are sweeping and overly general. Many of the statements are also false. For example, the epidemiology of acute febrile illness in sub-Saharan Africa is comparatively well characterized – even though non-malarial etiologies are not. There's really no meaningful difference between diagnosis of febrile illness in developing African countries versus more developed countries – virtually all acute fevers in both settings are treated empirically and without a laboratory diagnosis, with the exception of malaria and COVID.

We have altered the text in the introduction. However, we maintain that the epidemiology of non-malarial acute febrile illness, which would include viral aetiologies is not as well characterised in Africa as it is in high income countries such as the United Kingdom. Diagnostic inequality in low and middle income countries such as Uganda is well described. There is a lack of the molecular diagnostic infrastructure that is required for clinical diagnosis of viral pathogens in much of Africa, and where such capacity exists it is highly centralised. This is in contrast to the broad diagnostic molecular diagnostic panels that exist in in developed countries and are available both in secondary and primary care.

Figure 1. The phylogenetic tree includes 16 members of the genus Ledantevirus. However, there are currently 21 recognized ledanteviruses. All should be included, especially given the emphasis of this paper on the viral genus and not just LDV. The full list of ledantevirus member species can be found in the ICTV ledantevirus chapter: https://eur03.safelinks.protection.outlook.com/?url=https%3A%2F%2Fictv.global%2Freport%2Fchapter%2Frhabdoviridae%2Frhabdoviridae%2Fledantevirus&data=05%7C02%7CJames.Shepherd.2%40glasgow.ac.uk%7Cf99323e631e24b7d7db908dc322da49e%7C6e725c29763a4f5081f22e254f0133c8%7C1%7C0%7C638440418819280857%7CUnknown%7CTWFpbGZsb3d8eyJWIjoiMC4wLjAwMDAiLCJQIjoiV2luMzIiLCJBTiI6Ik1haWwiLCJXVCI6Mn0%3D%7C0%7C%7C%7C&sdata=lv3yWocOUnUnhKi6hWpdO7OBhVqhu6v6bh8h3o743lk%3D&reserved=0. The authors will note that coding complete genomes are available for all 20 currently recognized member species and for Tongren rhabd tick virus 2, which is currently unclassified but which is also a ledantevirus and should be included too. This chapter also includes very useful summary information about the natural history of these viruses (see "Biology" subheading). This would be a good (although not complete) starting point for the literature review mentioned above.

We have updated the phylogenetic tree in Figure 1 as suggested.

Figure 1. Similar to the above comment, there are 32 recognized alpharhabdovirine genera, but only 20 are included in the tree (see here: https://eur03.safelinks.protection.outlook.com/?url=https%3A%2F%2Fictv.global%2Freport%2Fchap

ter%2Frhabdoviridae%2Frhabdoviridae&data=05%7C02%7CJames.Shepherd.2%40glasgow.ac.uk%7Cf99323e631e24b7d7db908dc322da49e%7C6e725c29763a4f5081f22e254f0133c8%7C1%7C0%7C638440418819288981%7CUnknown%7CTWFpbGZsb3d8eyJWIjoiMC4wLjAwMDAiLCJQIjoiV2luMzIiLCJBTiI6Ik1haWwiLCJXVCI6Mn0%3D%7C0%7C%7C%7C&sdata=GQ8ZuT%2FVhyf7rhn2KOCf1uKBAnjZYV%2Bag9tozo85TqQ%3D&reserved=0). However, the other alpharhabdovirine genera do not add to the story presented here, and in fact the addition of outgroups reduces phylogenetic resolution. The tree would be more informative with a single outgroup – I would suggest vesicular stomatitis Indiana virus as the exemplar species of the genus Vesiculovirus. Thus, a new tree should consist of all current members of the Ledantevirus genus and VSIV as the outgroup.

We have updated the phylogenetic tree in Figure 1 as suggested.

Line 179. Biases associated with including only women of childbearing age in the serosurvey should be discussed fully in the Discussion. This is a major limitation affecting the validity of the study, and it does not adhere to the human subjects policies of the NIH regarding sex and age representation.

We have added text discussing the limitations of our use of an all-female cohort in the discussion.

Lines 263-274 and 682-687. It is unclear why these particular environmental variables were assessed, based on current knowledge about the ecology and epidemiology of the ledanteviruses. The authors should re-do this analysis using variables that are carefully chosen to test hypotheses about the reservoirs and transmission of ledanteviruses. For example, such variables could include: bat density (or proxy measures such as the proximity of caves); small mammal biodiversity; the density of reported acute fevers of unknown origin, etc. These sorts of ecological analyses can be informative, but only if the variables are carefully selected to test highly specific hypotheses about modes of transmission. Otherwise, as is the case currently, the analyses tend to uncover broad factors that are difficult to interpret because they are non-specific and confounded.

The variables were selected based on freely available datasets comprising environmental and livestock density data. Unfortunately, accurate data for the suggested additional variables such as bat density and small mammal biodiversity was not readily available to us and to collect such data would be beyond the resource of the present study. We agree that an analysis incorporating these variables should be combined with further

seroprevalence studies comprising prospectively recruited nationally representative population samples combined with more extensive individual-level metadata.

Line 278. “Environmental” is confusing because it sounds as if the virus was floating in a lake or found on a log. Change to “A ledantevirus in a wild rodent.” See next comment.

We have re-written the text in this heading.

Lines 279-303. The finding of fragmentary ledantevirus genetic material in fewer than 1% of captured rodents is very similar to what has been found for other ledanteviruses and other rhabdoviruses. The likely explanation is that the virus was not, in fact, infecting the mouse, but rather that it was infecting an insect with which the mouse interacted. This might have been an ectoparasite of the mouse (e.g. a flea, louse, mite, etc). Carriage of ledanteviruses in ectoparasites is a central feature of the natural history of the ledanteviruses. The presence of fragmentary viral RNA in the blood of the mouse would thus indicate transient or low-level infection. It is equally likely that the mouse merely ingested an ectoparasite (“allogrooming”) or ate a non-parasitic invertebrate infected by the virus. In the latter case, viral RNA appeared in the blood of the mouse because oral ingestion of viruses leads to viral genetic material in blood in small mammals (this is documented for many viruses, including rhabdoviruses and ledanteviruses). Concluding that the rodent is infected from the data available would repeat a common error made in the study of these and other viruses. This is an example of why a careful and comprehensive review of the literature would be necessary for proper interpretation of data in this study.

We disagree with the suggestion that the most likely explanation is that the viral RNA detected in the serum of this rodent likely the result of ingestion of an infected arthropod. All other phylogroup B ledanteviruses currently described have been demonstrated to cause infection of vertebrates, as evidenced by isolation of live virus from tissue or serum samples. Vaprio virus was isolated from homogenate of lung and heart from a bat. Keuraliba virus was isolated from the liver of a gerbil. Kern Canyon virus was isolated from homogenate of heart and spleen from a bat. Le Dantec virus has been isolated from human blood, is associated with a clinical syndrome of acute fever. Le Dantec virus infection has been demonstrated to lead to development of neutralising antibody. It is therefore reasonable to infer that the detection of the near-complete genome of a putative phylogroup B ledantevirus from the blood of a rodent in this case represents infection rather than fragmentary RNA related to a meal. We will revise the text to reflect this possibility but disagree that this is the most likely explanation based on the information available.

Figure 6. The new virus does not meet the ICTV species demarcation criteria for the genus Ledantevirus: https://eur03.safelinks.protection.outlook.com/?url=https%3A%2F%2Fictv.global%2Freport%2Fchapter%2Frhabdoviridae%2Frhabdoviridae%2Fledantevirus&data=05%7C02%7CJames.Shepherd.2%40glasgow.ac.uk%7Cf99323e631e24b7d7db908dc322da49e%7C6e725c29763a4f5081f22e254f0133c8%7C1%7C0%7C638440418819292284%7CUnknown%7CTWFpbGZsb3d8eyJWIjoiMC4wLjAwMDAiLCJQIjoiV2luMzIiLCJBTiI6Ik1haWwiLCJXVCI6Mn0%3D%7C0%7C%7C%7C&sdata=LzCoHOqwNE3WS%2BcpGM9ysMmnuOgH%2BE0kkyQGhQt0EOg%3D&reserved=0. In other words, it may be a variant of LDV or KEUV and not a novel virus at all. The authors should delete any text referring to this as a novel virus throughout the manuscript. They should give the virus a placeholder name such as “Ledantevirus from Mastomys erythroleucus” but not use the name “Odro virus” or any other such label. This is important because of the great difficulty of un-doing the erroneous assignment of novel viral taxon names, which is unfortunately pervasive and leads to endless confusion in viral taxonomy. In the Discussion, the authors should mention the species demarcation criteria for the Ledanteviruses and state that more complete genome sequencing would be required to ascertain whether this virus is a putative new member species of the genus or a variant of a currently recognized member species. If, in the future, the authors do indeed generate such sequence data, they should submit a Taxonomic Proposal to ICTV, who will then evaluate whether the virus merits assignment as a novel species.

> ***As suggested, we have removed reference to “Odro virus” or reference to it as a novel virus and termed it “Mastomys erythroleucus*** **- associated ledantevirus”** ***We agree that the complete genome is required to determine whether this virus represents a novel virus. However, based on the genomic information available, which is approximately 90% complete for the polymerase, and 72% complete for the glycoprotein, this appears likely to be the case. Amino acid divergence is 18.4% in the L protein and 24.1% in the G protein – clearly exceeding the thresholds set by the ICTV (7% divergence in L and 15% divergence in G).***

Line 340-342. It is fascinating that the authors found LDV in an East African patient, and the serological data are intriguing. These data could be very useful and interesting for the field.

Lines 342-344. Delete, as the data do not support these conclusions.

> ***We have deleted the text as above.***

Line 349-350. Another early case of LDV involved a British man who became infected whilst working as a labrourer at a shipyard in which he was bitten by an insect on a boat that had arrived from Africa (apologies; I cannot remember the reference). This is part of the evidence that LDV is vector-borne.

We have now referred to this study in the manuscript. The patient in this case (Woodruff et al 1977) was diagnosed with Le Dantec virus infection several years following his presentation based on serology (complement fixation). No virus was isolated from the patient. As demonstrated in our manuscript, the serological diagnosis of viral infection is often nonspecific and may be related to cross-reactivity, hence our caution in attributing the presentation of the case described in 1977 to LDV. Additionally, at the time of publication, Le Dantec virus had only been identified in West Africa, hence the inference that the patient had acquired the infection from the insect bite he sustained whilst unloading a ship from Nigeria. Our findings show that ledanteviruses, including LDV are present in East Africa, indicating that the positive serology in this patient may also reflect an infection acquired during his time in East Africa (he had reportedly served in the British army in Sudan).

Lines 346-356. The current consensus is that all ledanteviruses are vector-borne and that humans are infected aberrantly. It is therefore not accurate to contrast the ledanteviruses with the sigmaviruses and vesiculoviruses, as the authors do in this paragraph. The ledanteviruses and vesiculoviruses likely have extremely similar ecologies, whereas the sigmaviruses are vertically transmitted amongst insects. The authors should re-write the discussion to emphinasize not the associated hosts with which each genus of virus has been discovered (which is biased by sampling) but rather the mode of transmission – for example, vertical in insects for sigmaviruses, horizontal among mammals for lyssaviruses, and vector-borne and vertical [in insects] for both the ledanteviruses and vesiculoviruses.

We agree that the comparison is inaccurate and have significantly rewritten the discussion.

Lines 374-376. Agreed, LDV is a pathogen when it infects humans.

Lines 377-379. Again, these results are discussed without adequate context. A Google Scholar search for "Uganda ledantevirus" reveals a number of studies not discussed or cited by the authors that describe ledanteviruses in precisely the region where they document high human seropositivity rates. The availability of a rich literature on ledanteviruses in this area certainly necessitates a careful and comprehensive review that thoughtfully places the current findings in the context of past research on these agents in this region (and beyond).

We have updated the discussion with a more extensive description of the ledanteviruses detected in Uganda and beyond.

Lines 382-390. These are sweeping statements that are not germane to this study. Delete. Focus instead on narrow discussion of the particular research findings, in appropriate context within the published literature.

We have significantly re-written the discussion.

Lines 394-411. Delete/rewrite, as explained elsewhere herein.

We have significantly re-written the discussion.

Lines 444-453. Delete. This cannot yet be called a novel virus, and the interpretation of the ecology of the virus as having a mammal reservoir is very likely incorrect (see comments above).

We have rewritten this section.

Lines 508-511. Bioinformatics methods are inadequate. Please add full details of quality assurance/control measures, such as Phred cutoffs, minimum read lengths, chimera reomoval, etc.

We have added details of read quality control to the methods section.

Reviewer #2: My report is attached as this box does not allow the use of italics.

This paper reports the detection by metagenomic sequencing of Le Dantec virus in a male child who had presented to a local health centre in Uganda with a febrile illness. Neutralising antibody to LDV was detected in serum from the patient, a close relative, as well as two other unrelated individuals from Uganda and an individual in the UK who had previously resided in Africa. A serological survey conducted across Uganda indicated widespread human infection to Le Dantec virus or related ledanteviruses, with regional variations in prevalence. A novel ledantevirus, named Odro virus, was also detected by metagenomic sequencing of blood from a rodent (Mastomys erythroleucus)

collected in the Arua district of northern Uganda. Major aspects of this study, including the detection of Le Dantec virus have been reported previously in a non-peer-reviewed paper and mentioned briefly in a published review by the same authors.

Overall, this manuscript is written very well and describes a well-constructed and informative study. I find no fault with the technical aspects of the paper, data presentation or the reporting and interpretation of the results.

I have only relatively minor issues that I would like to see addressed, particularly the poor use of approved taxonomic nomenclature which requires revision throughout.

1. The author's fail to acknowledge a previous report of putative Le Dantec virus infection in a dock worker from Wales who had not been out of Britain for 20 years but had been bitten by an insect in 1969 whilst unloading a ship from Nigeria. (Woodruff et al BMJ 1977). Although evidence of this infection was based only on CF antibody against Le Dantec virus, it should be noted in the paper.

We have now referenced this paper in the manuscript. Interestingly, the patient had previously served in the British army in East Africa (Sudan, Eritrea and Egypt). At the time of publication Le Dantec virus had only been identified in West Africa, hence the inference that the patient had acquired the infection from the insect bite he sustained whilst unloading a ship from Nigeria. Our findings now show that ledanteviruses are, at least recently, present in East Africa. The positive serology in this patient may thus reflect an infection acquired during his time in East Africa.

2. The original description of the isolation of Le Dantec virus was in 1968 in a report from Paul Brès of the Reference Centre for Arboviruses at the Pasteur Institute in Dakar. This report is in French and somewhat difficult to access but it is referenced in the International Catalogue of Arboviruses (Berge, 1975) which is now curated online by the CDC. It would be appropriate to make some reference to this original report in addition to the secondary report of Cropp et al (1985) which has been cited by the authors.

We have referred to the original report.

3. Ledanteviruses of phylogroup B differ from members of other phylogroups in that they include a small ORF (U1) in an additional transcriptional unit between the G and L genes. It would be useful if the authors indicated whether this ORF was detected in the genome sequence of Odro virus.

Unfortunately this region of the genome has not been completely covered in our sequencing. There is however an open reading frame present immediately before the initiation of the L ORF. This is separated from the L ORF by an intergenic region consistent with that of other ledanteviruses. The pre-L ORF has no clear homology to the U1 proteins of other rhabdoviruses, however there is also no homology to glycoproteins of ledanteviruses, indicating that that this region likely represents the U1 ORF of this virus. We have added details of this to the text of the manuscript.

4. The authors refer (line 366) to two novel orthobunyaviruses from Uganda that have been associated with febrile illness. One wonders why the authors do not refer to multiple tibroviruses (family Rhabdoviridae) that have also been associated with febrile illness in central Africa. Bas Congo virus from the Democratic Republic of Congo (Grard et al PLoS Path 2012), Mundri virus from South Sudan (Edridge et al Viruses 2016), Ekpoma virus 1 from Nigeria and Ekpoma virus 2 from Nigeria and Angola (Stremlau et al PLoS NTD 2015; Kuhn et al Viruses 2020) have each been detected by NGS in humans, in some cases in association with febrile illness. The difficulties in establishing causal relationship for Bas Congo virus and other such viruses identified by metagenomic sequencing has also been discussed previously by these and other authors. Reference to human infection in central Africa with these other rhabdoviruses would be a useful addition to the discussion.

We agree that discussion of the tibroviruses detected by metagenomic sequencing is highly relevant to this study and we have expanded upon this in the discussion.

5. Taxonomic nomenclature is expressed incorrectly throughout the manuscript. Guidelines for correct taxonomic terminology and usage are described in detail on the ICTV website (https://ictv.global/filebrowser/download/440) and elsewhere (Zerbini et al Arch Virol 167:1232, 2022; Walker et al Animals 12:1363, 2022).

Line 18 The authors state “Le Dantec virus (LDV), type species of the genus Ledantevirus…”. Firstly, ICTV no longer recognises type species but instead user exemplar members of a species. Secondly, viruses are not species but are assigned as members to a species. Le Dantec virus is assigned taxonomically as a member of the species Ledantevirus ledantec, genus Ledantevirus, family Rhabdoviridae. A paper describing the difference between viruses (concrete entities) and virus species (abstract entities of human construction) can be found on the ICTV website.

This would be correctly expressed as “Ledantec virus (LDV), assigned to the species Ledantevirus ledantec, genus Ledantevirus, family Rhabdoviridae…..”

Line 25 Should be “…was confined to ledanteviruses,…” (no caps , no italics).

Line 28 Should be “Ledantevirus infection…” (no italics – caps only as the start of a sentence).

Line 77 Should be “…detection of a novel ledantevirus,..” (no caps , no italics)

Line 105 Correctly expressed (caps and italics) as this refers to the taxon, not the virus(es).

Line 287 Should be “..mapped to viruses of different species within the genus Ledantevirus by blastx.”

Line 288 Should be “..mapped to ledanteviruses.” (no caps, no italics)

Line 291 Should be “..mapped to members of the genus Ledantevirus.” (caps and italics)

Line 295 Should be “…of the genus Ledantevirus,..”

Line 296-7 Should be “…suggest Odro virus can be assigned to a novel species within the genus Ledantevirus”

Line 301 Correctly expressed (no caps and no italics) as this refers to the viruses, not the taxon.

Line 317 Should be “…members of the genus Ledantevirus,..”

Line 345 Should be “…members of the genus are divided into three phylogroups” (phylogroups are not taxa but are phylogenetic clusters of viruses).

Line 351 Should be “…members of the genus Ledantevirus,..”

Line 359 Should be “…viruses of different species detected by sequencing..” (viruses [concrete entities] can be sequenced but species [abstract entities] cannot be sequenced)

Line 379/80 Should be “…members of the genus Ledantevirus,..”

Line 401 Should be “genera Taterea and Taterillus.”

Line 406,444 Should be “…ledantevirus..” (no caps, no italics)

Line 503 Should be “…ledanteviruses…” (no caps, no italics)

Line 515,516 Should be “…ledantevirus..” (no caps, no italics)

Line 529 Should be “..the Alpharhabdovirinae..”

Line 532 Should be “…members of the genus Ledantevirus,..”

We have adjusted the taxonomic nomenclature as suggested.

Reviewer #3: The manuscript by Shepherd etal provides a much needed, albeit retrospective, serosurvey for the burden of exposure to Ledantec group of viruses in Western Uganda. While the virus was isolated from a human 40 years ago, to date there is little know about its impact on human health across Africa. Shepherd etal provides evidence of wide seroprevalence among humans and certainly in an areas far away from its initial discovery in Senegal, as well as evidence of a new virus in the species present in rodents. While this study is illuminating and of value to the wider scientific community there are a number of minor issues that could benefit from the authors' attention.

Abstract:

please use the updated ICTV nomenclature. it is species: Ledantevirus ledantec

We have made the suggested updates to the text.

Results:

neutralization assays based on the use of pseudotypes are less specific. Given that the investigators received a viral sample of the Senegal isolate, have the PRNT assay been performed for comparison? The fact that there is crosssreactivity is not surprising at all, suggesting as the authors indicated that whatever is being detected is a member virus of the species Ledantevirus ledantec

Unfortunately, we were not able to perform PRNT assay on the isolate, and we have discussed potential limitations of the use of pseudotypes in the manuscript. However, we believe that the use of pseudotypes in this context is appropriate. Pseudotype virus neutralisation assays have been shown to correlate well with authentic virus neutralisation assays.

Cantoni D, Wilkie C, Bentley EM, Mayora-Neto M, Wright E, Scott S, Ray S, Castillo-Olivares J, Heeney JL, Mattiuzzo G, Temperton NJ. Correlation between pseudotyped virus and authentic virus neutralisation assays, a systematic review and meta-analysis of the literature. Front Immunol. 2023 Sep 18;14:1184362. doi: 10.3389/fimmu.2023.118436

Pseudotype-based approaches have also recently been employed to investigate population exposure to other rhabdoviruses, including Bas-Congo virus:

Munyeku-Bazitama et al. "Seroprevalence of Bas-Congo virus in Mangala, Democratic Republic of the Congo: a population-based cross-sectional study." The Lancet. Microbe, S2666-5247(24)00021-1. 28 Mar. 2024, doi:10.1016/S2666-5247(24)00021-1

Discussion:

line 344-5: the 2022 report is already outdated. Currently there are 20 identified members within the species Ledantevirus ledantec. Consider citing the ICTV webpage (https://eur03.safelinks.protection.outlook.com/?url=https%3A%2F%2Fictv.global%2Freport%2Fchapter%2Frhabdoviridae%2Ftaxonomy%2Frhabdoviridae&data=05%7C02%7CJames.Shepherd.2%40glasgow.ac.uk%7Cf99323e631e24b7d7db908dc322da49e%7C6e725c29763a4f5081f22e254f0133c8%7C1%7C0%7C638440418819297225%7CUnknown%7CTWFpbGZsb3d8eyJWIjoiMC4wLjAwMDAiLCJQIjoiV2luMzIiLCJBTiI6Ik1haWwiLCJXVCI6Mn0%3D%7C0%7C%7C%7C&sdata=DusOX0idcKIQRHBVjrGDmV2JIoDkpacu6p2WhtV8TfM%3D&reserved=0)

We have updated the citation as suggested.

line 373. replace "classed' with 'classified'

We have made the suggested changes to the text.

line 375-6. were the intent was to say '...unavailability of samples from the convalescent phase of the illness to demonstrate seroconversion'

We had insufficient serum sample from the acute phase of the patients illness, so all serological testing was performed on convalescent samples collected 4 weeks following recruitment rather than from their initial presentation. Thus we were unable to demonstrate a rise in titre between the point of recruitment and the convalescent phase which would have been highly supportive of acute infection with Le Dantec virus as the cause of the patients illness.

line 441. The statement is contrary to what the authors indicated so far. suggest remove "ldv" and replace as "...we identified members of the species Ledantevirus ledantec as the likely causative..."

We demonstrated infection by Ledantevirus ledantec as the likely causative agent of the acute illness experienced by the index patient by mNGS and serology. Our population level serological data indicates that both Ledantevirus ledantec and other phylogroup B ledanteviruses are causing human infection in Uganda.

Methods:

line 458-9. include the approval dates of the mentioned ethics protocols. specify whichh protocols were for human sampling and which for the rodent collections

We have added approval dates and indicated the protocols for human and animal sampling in the text.
