# Supplementary material for: Widespread human exposure to ledanteviruses in Uganda: A population study
Source: PLoS Negl Trop Dis. 2024 Jul 8;18(7):e0012297. doi: 10.1371/journal.pntd.0012297 (PMC11257405; doi:10.1371/journal.pntd.0012297)
Supplement: Attachment — Submitted filename: PNTD-D-23-01521.docx [file pntd.0012297.s016.docx]

This paper reports the detection by metagenomic sequencing of Le Dantec virus in a male child who had presented to a local health centre in Uganda with a febrile illness. Neutralising antibody to LDV was detected in serum from the patient, a close relative, as well as two other unrelated individuals from Uganda and an individual in the UK who had previously resided in Africa. A serological survey conducted across Uganda indicated widespread human infection to Le Dantec virus or related ledanteviruses, with regional variations in prevalence. A novel ledantevirus, named Odro virus, was also detected by metagenomic sequencing of blood from a rodent (*Mastomys erythroleucus*) collected in the Arua district of northern Uganda. Major aspects of this study, including the detection of Le Dantec virus have been reported previously in a non-peer-reviewed paper and mentioned briefly in a published review by the same authors.

Overall, this manuscript is written very well and describes a well-constructed and informative study. I find no fault with the technical aspects of the paper, data presentation or the reporting and interpretation of the results.

I have only relatively minor issues that I would like to see addressed, particularly the poor use of approved taxonomic nomenclature which requires revision throughout.

1. The author’s fail to acknowledge a previous report of putative Le Dantec virus infection in a dock worker from Wales who had not been out of Britain for 20 years but had been bitten by an insect in 1969 whilst unloading a ship from Nigeria. (Woodruff et al BMJ 1977). Although evidence of this infection was based only on CF antibody against Le Dantec virus, it should be noted in the paper.
2. The original description of the isolation of Le Dantec virus was in 1968 in a report from Paul Brès of the Reference Centre for Arboviruses at the Pasteur Institute in Dakar. This report is in French and somewhat difficult to access but it is referenced in the International Catalogue of Arboviruses (Berge, 1975) which is now curated online by the CDC. It would be appropriate to make some reference to this original report in addition to the secondary report of Cropp et al (1985) which has been cited by the authors.
3. Ledanteviruses of phylogroup B differ from members of other phylogroups in that they include a small ORF (U1) in an additional transcriptional unit between the *G* and *L* genes. It would be useful if the authors indicated whether this ORF was detected in the genome sequence of Odro virus.
4. The authors refer (line 366) to two novel orthobunyaviruses from Uganda that have been associated with febrile illness. One wonders why the authors do not refer to multiple tibroviruses (family *Rhabdoviridae*) that have also been associated with febrile illness in central Africa. Bas Congo virus from the Democratic Republic of Congo (Grard et al PLoS Path 2012), Mundri virus from South Sudan (Edridge et al Viruses 2016), Ekpoma virus 1 from Nigeria and Ekpoma virus 2 from Nigeria and Angola (Stremlau et al PLoS NTD 2015; Kuhn et al Viruses 2020) have each been detected by NGS in humans, in some cases in association with febrile illness. The difficulties in establishing causal relationship for Bas Congo virus and other such viruses identified by metagenomic sequencing has also been discussed previously by these and other authors. Reference to human infection in central Africa with these other rhabdoviruses would be a useful addition to the discussion.
5. Taxonomic nomenclature is expressed incorrectly throughout the manuscript. Guidelines for correct taxonomic terminology and usage are described in detail on the ICTV website (<https://ictv.global/filebrowser/download/440>) and elsewhere (Zerbini et al Arch Virol 167:1232, 2022; Walker et al Animals 12:1363, 2022).

Line 18	The authors state “Le Dantec virus (LDV), type species of the genus *Ledantevirus*…”. Firstly, ICTV no longer recognises type species but instead user exemplar members of a species. Secondly, viruses are not species but are assigned as members to a species. Le Dantec virus is assigned taxonomically as a member of the species *Ledantevirus* *ledantec*, genus *Ledantevirus*, family *Rhabdoviridae*. A paper describing the difference between viruses (concrete entities) and virus species (abstract entities of human construction) can be found on the ICTV website.

This would be correctly expressed as “Ledantec virus (LDV), assigned to the species *Ledantevirus ledantec*, genus *Ledantevirus*, family *Rhabdoviridae*…..”

Line 25	Should be “…was confined to ledanteviruses,…” (no caps , no italics).

Line 28	Should be “Ledantevirus infection…” (no italics – caps only as the start of a sentence).

Line 77	Should be “…detection of a novel ledantevirus,..” (no caps , no italics)

Line 105	Correctly expressed (caps and italics) as this refers to the taxon, not the virus(es).

Line 287	Should be “..mapped to viruses of different species within the genus *Ledantevirus* by blastx.”

Line 288	Should be “..mapped to ledanteviruses.” (no caps, no italics)

Line 291	Should be “..mapped to members of the genus *Ledantevirus*.” (caps and italics)

Line 295	Should be “…of the genus *Ledantevirus*,..”

Line 296-7	Should be “…suggest Odro virus can be assigned to a novel species within the genus *Ledantevirus*”

Line 301	Correctly expressed (no caps and no italics) as this refers to the viruses, not the taxon.

Line 317	Should be “…members of the genus *Ledantevirus*,..”

Line 345	Should be “…members of the genus are divided into three phylogroups” (phylogroups are not taxa but are phylogenetic clusters of viruses).

Line 351	Should be “…members of the genus *Ledantevirus*,..”

Line 359	Should be “…viruses of different species detected by sequencing..” (viruses [concrete entities] can be sequenced but species [abstract entities] cannot be sequenced)

Line 379/80	Should be “…members of the genus *Ledantevirus*,..”

Line 401	Should be “genera *Taterea* and *Taterillus*.”

Line 406,444	Should be “…ledantevirus..” (no caps, no italics)

Line 503	Should be “…ledanteviruses…” (no caps, no italics)

Line 515,516	Should be “…ledantevirus..” (no caps, no italics)

Line 529	Should be “..the *Alpharhabdovirinae*..”

Line 532	Should be “…members of the genus *Ledantevirus*,..”
