# Supplementary material for: Widespread human exposure to ledanteviruses in Uganda: A population study
Source: PLoS Negl Trop Dis. 2024 Jul 8;18(7):e0012297. doi: 10.1371/journal.pntd.0012297 (PMC11257405; doi:10.1371/journal.pntd.0012297)
Supplement: Attachment — Submitted filename: revision_letter2.docx [file pntd.0012297.s018.docx]

9^th^ June 2024

Dear colleagues,

**Re: Widespread human exposure to ledanteviruses in Uganda: a population study.**

Many thanks for your invitation to submit a revised version of our manuscript. We have addressed the minor revisions suggested by the reviewers. Please find below a point-by-point response to the comments made by the reviewers overleaf.

Yours faithfully,

Dr James Shepherd

Professor Emma Thomson

MRC-University of Glasgow Centre for Virus Research

Reviewer #1: Many thanks to the authors for their careful consideration of all the reviewer comments and for re-writing sections of the text. The review of ledanteviruses in Uganda and elsewhere is good. I have no major suggested further changes. Please consider the following minor changes.

Line 34. Add “(Rhabodoviridae)” in italics after “Ledantevirus.” This will make the author summary stand alone for those who may not realize that Ledantevirus is a rhabdoviral genus.

**We have made the suggested changes as above.**

Line 58. Check with journal as to whether “Next Generation Sequencing” should be capitalized. None of the words are proper nouns.

**We will liaise with the journal regarding the correct capitalization during the publication process.**

Line 237. It’s not entirely clear what “spatial restriction” means. Consider an alternative term.

**We have altered the text in this paragraph to *“There was no obvious pattern consistent with geographical restriction of sera with higher IC50 to either LDV or KEUV.”.***

Lines 271-302. This is a long paragraph and it’s difficult to digest all the information as a block. Consider splitting it into two shorter paragraphs, for example with a paragraph break at line 287 and some minor corresponding rearrangements of text.

**We have rearranged the paragraph to provide a natural break as suggested.**

Line 374. The Hippoboscidae are actually not bat flies. They are “louse flies” or “keds.” Bat flies are in different families. Change to “hippoboscid fly.”

**We have made the suggested changes as above.**

Line 406. Change “female” to “woman” because, following the previous sentence, it sounds as if a female bat presented to hospital.

**We have replaced female to “girl”.**

Lines 415-449. This is again a very long paragraph. There is a natural paragraph break at line 428, if a transition statement such as “In illustration of these difficulties” were added before “Several novel rhabdoviruses.”

**We have rearranged the paragraph to provide a natural break as suggested.**

Line 451. The word “probable” may be too cautious here. The evidence is quite strong of LDV being a pathogen in humans (but not bats or arthropods). So, remove “probable” and change line 451 to “a pathogen in humans.”

**We have made the suggested changes as above.**

Line 518. Consider changing “framework” to “relationship.”

**We have made the suggested changes as above.**

Reviewer #2: line 1023 - a species (abstraction) cannot cause illness. Only the virus (animate) can cause illness. Should read: "We identified Le Dantec virus (species Ledantevirus ledantec [italics]) as the likely causative agent...."

**We have made the suggested changes as above.**
